# Trade-Off between Facilitation and Interference of Allelopathic Compounds in Vegetation Recovery: The Case of *Rosmarinus officinalis* in Degraded Gypsum Habitats

**DOI:** 10.3390/plants11030459

**Published:** 2022-02-07

**Authors:** Helena García-Robles, Eva María Cañadas, Juan Lorite, Emilia Fernández-Ondoño

**Affiliations:** 1 Departamento de Botánica, Universidad de Granada, Campus Fuentenueva s/n, 18071 Granada, Spain; ecanadas@ugr.es (E.M.C.); jlorite@ugr.es (J.L.); 2 Departamento de Edafología y Química Agrícola, Universidad de Granada, Campus Fuentenueva s/n, 18071 Granada, Spain; efernand@ugr.es

**Keywords:** mining restoration, allelopathic compounds, essential oils, facilitation

## Abstract

*Rosmarinus officinalis* advantageously competes with other species in restored gypsum outcrops*,* and further research is needed to understand the causes. Specifically, we focus on the potential allelopathic effects derived from its terpenes on the emergence of gypsum species. To this end, we established 120 circular subplots in a previously restored gypsum outcrop, and randomly applied four different treatments based on the presence/absence of rosemary plants and their leaves on the soil. Afterwards, we conducted an experimental sowing of native gypsophiles. All subplots were monitored to estimate seedling emergence, and soil and leaf samples were analysed for terpenes. The results show that the treatments had significant effects on the overall emergence of seedlings, and terpenes were found in rosemary leaves and soils, with no significant differences in terpene composition. In particular, we identified a clear negative effect in the treatment where rosemary plants were eliminated but its leaves were left along with allelopathy (2.57 ± 0.54 individuals/subplot). Unexpectedly, the presence of rosemary plants seems to facilitate the emergence of gypsum species (9.93 ± 1.61 individuals/subplot), counteracting the effects of the allelopathic substances in the soil. Consequently, we do not suggest removing rosemary plants in early stages to encourage the emergence of gypsum species in restored areas.

## 1. Introduction

Gypsum habitats are spread across the world and comprise approximately 100 million hectares [1]. Despite the fact that they are sparsely represented all over the world, their importance is crucial. Covering 35,487 km^2^, the Iberian Peninsula boasts the largest area of gypsum outcrops in Europe [2]. They harbour an exceptionally adapted flora, which is unique within the European continent [3,4]. The peculiar edaphic characteristics of gypsum habitats define and determine the flora inhabiting them. This is the reason why there are so many endemic and native species in these particular areas, the so-called “gypsum species” as they shall be hereafter referred to as, which can be divided into “gypsophiles” and “gypsovags”. In this context, “gypsophiles” have been defined as those plant species restricted to gypsum soils, and “gypsovags” as those plant species commonly occurring on both gypsum and non-gypsum substrates [5].

Despite gypsum habitats being considered a priority for conservation [6], large gypsum areas and, thus, gypsum flora are disturbed by quarrying [7,8,9] and represent a major challenge in the restoration of disturbed singular environments [10]. In particular, the Iberian gypsum vegetation is being severely damaged since Spain is one of the main gypsum producers worldwide [11]. On this basis, previous works have demonstrated the limitations that natural succession has for the recovery of gypsum vegetation over the short or medium term, i.e., [7,8,12,13]. Consequently, there is a need to develop specific measures to restore these environments.

The recovery of gypsum areas has been satisfactorily approached through different techniques [14,15,16,17,18]. Particularly, in a former experiment, Ballesteros et al. [8] found a balanced emergence, survival, and growth in different substrate mixtures for the sown gypsum species (both gypsophiles and gypsovags). One of the sown gypsovags in that experiment was rosemary (*Rosmarinus officinalis* L.), which twelve years later presented a significantly higher coverage in all the treatments applied compared with the other sown species [19]. Rosemary is a Mediterranean native species characterised by a high tolerance to heat, drought, and to poor, dry, sandy, and rocky soil types [20]. From a phytochemical point of view, and similarly to other species of the Lamiaceae family [20,21], rosemary is characterised by the presence of essential oils with allelopathic properties, which could explain its competitive success against other species. Allelopathy is the process in which plants release phytotoxic compounds into the environment to inhibit the germination and growth of other plants sharing the same habitat [22]. Consequently, allelopathy plays a significant role in plant dominance, succession, species diversification, and the establishment of plant communities [23,24,25,26,27]. Wardle et al. [28] suggested that allelopathic inhibition may be more likely to occur in communities with low species richness, such as the gypsum habitat in the study area, in which soil biochemistry is likely to be determined by dominant plant species. In this sense, rosemary essential oils have been proven to inhibit or reduce seed germination (a key stage in plant development) for some weeds and crops such as *Silybum marianum* (L.) Gaertn or *Raphanus sativus* L. [21,29], which is the reason for which this species has been proposed as a bioherbicide to control seed germination [20,29,30]. Essential oils are a complex mixture of terpenes and other aromatic and aliphatic compounds in different proportions [31], whose potential synergic interactions are not yet well known [32]. Nonetheless, allelopathy is commonly described for individual terpenes in the bibliography [32]. For instance, α- and β-pinene, camphor, borneol, carvacrol, thymol, eucalyptol (or 1–8 cineole), limonene, o-cymenone, citronellol, camphene, α-phellandrene, and p-cymene have been extensively described as emergence inhibitors or retarders [20,21,33,34,35]. With regard to the inhibitory effect that *R. officinalis* essential oils have on the germination of some plants such as *S. marianum* and *R. sativus* [21,29], monoterpenes and sesquiterpenes [36], such as eucalyptol and borneol, are the main responsible compounds [20,21]. Moreover, Scrivanti et al. [37] reported that monoterpene transfer to the soil is higher in hot and dry climates, acting as important allelopathic agents. Considering all the above, *R. officinalis* could limit the recovery of degraded gypsum habitats in which germination is a determining stage due to the harsh edaphic conditions [5,10].

Consequently, in this study, we aimed to explore the potential interference of rosemary in the emergence of other gypsum species such as *Ononis tridentata* L. subsp. *crassifolia* (Dufour ex Boiss.) Nyman, *Helianthemum squamatum* (L.) Dum. Cours and *Lepidium subulatum* L., focusing on the role of its allelopathic compounds. Finally, based on the results, we seek to contribute to the enhancement of restoration plans for gypsum habitats, which are a priority for conservation.

## 2. Results

### 2.1. Effects of Treatments on Seedling Emergence

#### 2.1.1. Overall Emergence

**Figure 1:**
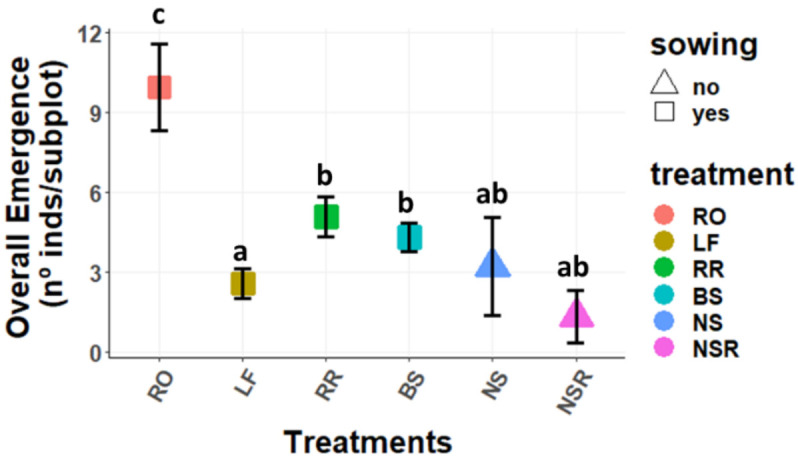
Mean (±SE) emergence of perennial species (nº of seedlings per subplot and treatment). Treatments: RO: *Rosmarinus officinalis*; LF: Litterfall; RR: Rosemary Removal; BS: Bare Soil; NS: non-sown subplots without rosemary; NSR: non-sown subplots with rosemary. Different letters represent statistically significant differences (*p* < 0.05) for the post hoc Tukey tests performed after the GLMMs, where “a” represents the lowest value and “c” the highest.

The overall emergence of perennial species as a group was rather low in the non-sown experimental areas (both with and without rosemary influence) and did not show significant differences from one another (Figure 1). Meanwhile, the treatments applied in the sown subplots indeed presented significant effects on the overall emergence of seedlings (Figure 1 and Supplementary Table S1). In particular, the “*Rosmarinus officinalis*” treatment registered the highest overall emergence (9.93 ± 1.61 individuals per subplot), with significant differences compared to the other treatments. On the other hand, the lowest overall emergence appeared in “Litterfall” treatment (2.57 ± 0.54 per subplot), which also had significant differences compared to the others. Eventually, the “Bare Soil” (4.30 ± 0.55 per subplot) and “Rosemary Removal” (5.07 ± 0.75 per subplot) treatments presented an intermediate overall emergence and no significant differences with each other.

**Figure 2:**
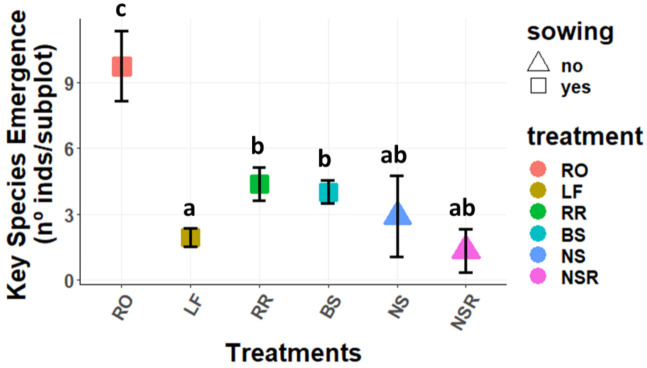
Mean (±SE) emergence of key species (nº of seedlings per subplot and treatment). Treatments: RO: *Rosmarinus officinalis*; LF: Litterfall; RR: Rosemary Removal; BS: Bare Soil; NS: non-sown subplots without rosemary; NSR: non-sown subplots with rosemary. Different letters represent statistically significant differences (*p* < 0.05) for the post hoc Tukey tests performed after the GLMMs, where “a” represents the lowest value and “c” the highest.

**Figure 3:**
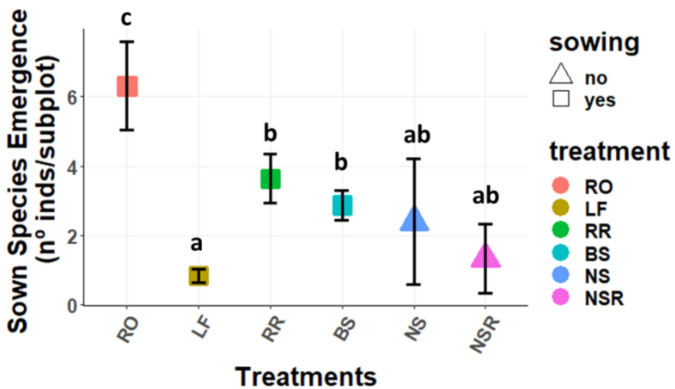
Mean (±SE) emergence of the sown species (nº of seedlings per subplot and treatment). Treatments: RO: *Rosmarinus officinalis*; LF: Litterfall; RR: Rosemary Removal; BS: Bare Soil; NS: non-sown subplots without rosemary; NSR: non-sown subplots with rosemary. Different letters represent statistically significant differences (*p* < 0.05) for the post hoc Tukey tests performed after the GLMMs, where “a” represents the lowest value and “c” the highest.

Focusing on the key species as a group (*Ononis tridentata* subsp. *crassifolia*, *Helianthemum squamatum*, *Lepidium subulatum*, *Thymus zygis* subsp. *gracilis*, *Rosmarinus officinalis*, *H. syriacum*, and *Stipa tenacissima*), or on the sown species as a group (*O. tridentata* subsp. *crassifolia*, *H. squamatum* and *L. subulatum*), the same pattern was found for emergence (Figure 2 and Figure 3, respectively).

#### 2.1.2. Emergence Per Species

Considering all the sown subplots, there has been little emergence of seedlings of *O. tridentata* subsp*. crassifolia* (14 individuals), *L. subulatum* (22 individuals), *S. tenacissima* (3 individuals), and *H. syriacum* (13 individuals), while a much higher emergence of *H. squamatum* (373 individuals) and *T. zygis* subsp. *gracilis* (137 individuals) seedlings occurred. In between, *R. officinalis* had an average emergence (52 seedlings).

Despite the generally low emergence registered for most of the species, multiple comparisons (Tukey’s test) showed significant differences between treatments for nearly all the key species (Table 1). More precisely, the “Bare Soil” treatment promoted the highest emergence with significant differences compared to the other treatments for *L. subulatum* and *H. syriacum*, whereas “*Rosmarinus officinalis*” was the most favourable treatment for *H. squamatum*, *R. officinalis* and *T. zygis* subsp. *gracilis*. In the case of *O. tridentata* subsp. *crassifolia* and *S. tenacissima*, emergence was equally low in all the treatments.

### 2.2. Essential Oils in Soils and Leaves

Chromatographic analyses of essential oils confirmed that there were monoterpenes and sesquiterpenes in leaves (GL and FL) and in the soils influenced by rosemary plants (RS). More precisely, a total of 39 terpenes were isolated (Table 2 and Supplementary Table S2), 10 of which are “key terpenes” with described allelopathy [20,21,33,34,35]. On the contrary, there were no terpenes in the bare soils (BS) (Table 2 and Supplementary Table S2).

The essential oil of green leaves presented at least 24 terpenes, and there were 23 in the fallen leaves, sharing a total of 18 different terpenes (Table 2). In the case of soil samples, there were no terpenes in the “Bare Soil”, whereas we found at least 30 different terpene compounds in the soils under rosemary plants (RS), among which 20 were also found in leaf samples (Table 2). According to the area (%) occupied by each terpene in the chromatographic curves (Supplementary Table S3), the most abundant terpenes in green leaves (more than 90% of the total) are α- and β-pinene, camphene, β-myrcene, limonene, eucalyptol, α-terpinene, terpinolene, camphor, borneol, bornyl acetate and caryophyllene, among which seven are “key terpenes”. In the case of fallen leaves, the most abundant terpenes (approximately 80–90% of the total) are α-pinene, camphene, p-cymene, eucalyptol, camphor, borneol, α-terpineol, caryophyllene, among which six of them are “key terpenes”. Finally, the most abundant terpenes in rosemary soil samples are α- and β-pinene, camphene, eucalyptol, camphor, borneol, verbenone, and caryophyllene, among which six are “key terpenes”.

Multiple comparisons after a multivariate analysis of the overall terpene content (Table 3) show significant differences between green leaves (GL) and fallen leaves (FL) and between green leaves (GL) and soils under rosemary plants (RS). Nonetheless, there are no significant differences between the terpene content in fallen leaves (FL) and soils under rosemary plants (RS).

Multiple comparisons between sample types per key terpenes after permutation tests for linear models are shown in Table 4. To highlight, significant differences were found between “BS” samples and the rest of the sample types for all key terpenes except for α-phellandrene and carvacrol, for which there were no significant differences between any sample types. In the case of p-cymene and limonene, there are marginal significant differences among “GL”, “FL”, and “RS”, and for β-pinene, there are significant differences between “GL” and “FL”.

## 3. Discussion

Results revealed clear significant effects of treatments on the general emergence of gypsum species (both gypsophiles and gypsovags), confirming the hypothesis that rosemary conditions the emergence of seedlings in gypsum areas (Table 5). In particular, we observed a clear negative effect of the “Litterfall” (LF) treatment, in which rosemary plants were eliminated but the leaves remained, which could be linked to the presence of allelopathic compounds.

According to the chromatographical analyses conducted on leaf and soil essential oils, there are four clear patterns regarding terpene composition: (1) rosemary-free soils rarely contain terpenes; (2) leaf samples, both green and fallen, contain terpenes with similar results to those found by Ormeño et al. [38] and Alipour et al. [39]; (3) soils somehow influenced by rosemary plants also contain terpenes; and (4) the samples of rosemary fallen leaves, rosemary green leaves and rosemary soils presented similar terpene compositions, characterised by the presence of terpenes previously described as emergence inhibitors or retarders, such as α- and β-pinene, camphene, p-cymene, limonene, eucalyptol, camphor and borneol [20,21,33,34,35]. Consequently, our results reveal there is a stream of terpenes from rosemary plants and leaves to the nearby soils, similarly to what Fisher [33] observed, suggesting a correlation between terpene presence and the inhibition of seedling emergence, especially for *O. tridentata* subsp. *crassifolia, L. subulatum, H. syriacum*, and *S. tenacissima*. Moreover, as a result of such terpene transfer, the sown subplots somehow influenced by rosemary may have similar terpene compositions, including those having undergone the “Rosemary Removal” treatment, in which terpenes may remain for a period of time after rosemary plant and fallen leaves removal [40].

In contrast to what could be expected, “*Rosmarinus officinalis*” treatment had a significant positive effect on the general emergence of perennial species despite its potentially high load of allelopathic terpenes. Furthermore, the highest emergence was found for this treatment, which made us consider the facilitation process as a determining factor in the early stages of plant emergence in stressful environments, more than allelopathy could be [41]. Regardless of their role as allelopathic compound producers, adult rosemary plants could offer shade and other benefits to the species emerging nearby (i.e., a safer regeneration of the micro-niche, higher water and nutrient availability, etc.), which has been proven to be of high relevance in degraded Mediterranean areas [42]. Actually, Pugnaire, and Luque [43] indicated that a combination of competition and facilitation effects often operates simultaneously among plant species in nature.

Interestingly, the lowest general emergence was registered for the “Litterfall” treatment. When rosemary plants were removed along with their facilitation effect, but rosemary fallen leaves remained, a strong inhibitory effect over the emergence of the key species was observed, probably due to the high content of allelopathic terpenes. Tormo-Blanes et al. [44] also detected that rosemary fallen leaves had negative effects on the emergence of perennial species when sowing was conducted on the litter layer. Scognamiglio et al. [27] found that the degradation of fallen leaves by microorganisms releases allelopathic substances, such as terpene compounds, which inhibits germination and/or plant development. In addition to this, Alias-Gallego et al. [40] reported that under arid conditions, allelopathic compounds are mainly released into the soil through the degradation of litter.

Rosemary-free soils, those with “Bare Soil” treatment, which are presumably drier, more compacted, nutrient-poor, rosemary terpenes-free, and receive direct insolation, showed a similar trend compared to soils having undergone the “Rosemary Removal” treatment, in which the rosemary plants and fallen leaves were removed for the experiment. Both presented lower emergence rates than the “*Rosmarinus officinalis*” treatment, but higher than those of the “Litterfall” treatment. The fact that emergence in the “Bare Soil” and “Rosemary Removal” treatments was usually comparable suggested that, if the source of terpene production (either rosemary plants or fallen leaves) was removed, monoterpenes (volatile fraction of essential oils) and sesquiterpenes would evaporate and decompose with time [45] and thus, the inhibiting effect toward other plant species would then cease.

Focusing on the sown species, there was little seedling emergence of *O. tridentata* subsp. *crassifolia* and *L. subulatum* in the experimental area, whereas a much higher emergence of *H. squamatum* occurred. The emergence of *O. tridentata* subsp. *crassifolia* was very low in all the treatments probably due to its commonly low germination rate (≤20%) [46,47], together with the low ratio of sown seeds for this species compared to the others. *L. subulatum* presented an especially low emergence rate in those treatments somehow influenced by a rosemary plant, whereas it found its peak of emergence in the “Bare Soil” treatment, suggesting that this species is especially sensitive to competition and allelopathy interference. Escudero et al. [48] observed that the seedlings of *L. subulatum* tend to establish on the gypsum surface crust and limit their habitat to a reduced niche beyond the scope of competitive interaction with other shrub species. Moreover, Escudero et al. [49] observed how the emergence of gypsophiles in gypsum soils could have been negatively affected by the allelopathic effects of some gypsovags. On the contrary, *H. squamatum* is able to grow in a field with a wide variety of soils, although its survival rate and growth are higher on genuine gypsum soils [50]. Notably, *H. squamatum* was the species which benefited the most from every treatment, especially from “*Rosmarinus officinalis*”. Foronda et al. [51] also observed the abundance of *H. squamatum* seedlings under the influence of a nurse species and in open areas. In addition to this, *H. squamatum* seems to be a strong competitor in gypsum habitats, since in the cases in which the emergence of this species followed an explosive trend, no seedlings of other species (or very few, as in “*Rosmarinus officinalis*” treatment) accompanied them, probably due to the scarcity of resources.

In the case of gypsovags, the response of seedling emergence to treatments was different for each species. It was generally low, except for *T. zygis* subsp. *gracilis*. For instance, *S. tenacissima* only emerged in “Rosemary Removal” treatment, which suggests that this species could have been negatively affected by shadowing (“*Rosmarinus officinalis*” and “Litterfall” treatments), as reported by García-Fayos and Gasque [52], and/or by physical barriers such as soil crust (“Bare Soil” treatment) [44]; moreover, its extremely low emergence in the “Rosemary Removal” treatment made us think that competition with *H. squamatum* and/or rosemary-induced allelopathy could have also affected this species. Although *H. syriacum* and *H. squamatum* usually coexist in gypsum habitats [53], after a period of severe drought, both populations may have been desynchronised [53], and *H. syriacum*, as a gypsovag species, may have been displaced by *H. squamatum,* which has proved to be a strong competitor under these circumstances. Despite its overall low emergence, *H. syriacum* seemed to be more favoured either by bare soils where there is no competition for resources, or by soils with rosemary plants, possibly due to its role as a nurse species. After *H. squamatum*, *T. zygis* subsp. *gracilis* was the key species with the best performance. This species emerged similarly in all the treatments except in “*Rosmarinus officinalis*”, where we found its highest emergence. *T. zygis* subsp. *gracilis* also produces essential oils with allelopathic effects [54] and should have the ability to selectively detect and respond to chemical signals [55]. Moreover, *T. zygis* subsp. *gracilis* seems to cope with competition very well [56], and as a gypsovag species emerging in gypsum habitats, it may have benefited from rosemary-induced facilitation [51]. Finally, *R. officinalis* has been observed to act as a diversity repellent [51]. In this sense, allelopathy could be a mechanism used by some gypsovags to avoid competition for resources and succeed in gypsum habitats [51]. Despite the great abundance of *R. officinalis* adult plants in the experimental area, seedling emergence for this species was rather low in treatments, except in “*Rosmarinus officinalis*”, where seed recruitment was effective, probably due to seed availability and facilitation processes. On top of this, there was one case in which rosemary seedlings emerged explosively in “*Rosmarinus officinalis*” treatment, hindering the emergence of other species’ seedlings, possibly as a result of the interdependence of resource competence and allelopathy [57]. Interestingly, in the “Rosemary Removal” and “Litterfall” treatments, emergence was very low even if these soils should contain a significant seed bank. Under these conditions, rosemary seedlings may have suffered from autotoxicity, as has been described for other allelopathic species [51,58].

Overall, restoration plans for degraded gypsum habitats should foster the development of species such as *O. tridentata* subsp. *crassifolia*, which could act as a nurse plant under these circumstances, as described for other species of the Fabaceae family [51], and thus help structure gypsum community. Moreover, restoration plans should avoid using allelopathic species [59].

## 4. Materials and Methods

### 4.1. Study Area

This study was conducted at a gypsum outcrop area in Escúzar, located in the southwestern area of Granada (Spain), 37°2′ N, 3°45′ W, at 950 m a.s.l., in the vicinity of an active gypsum quarry. The area is characterised by a continental Mediterranean climate, with moderately cold winters, hot summers, and a 4-month dry period with water deficit. The annual average temperature is 15 °C; the coolest average temperature, 7.6 °C, is reached in January, while the hottest average temperature, 24.2 °C, is reached in August. The average annual precipitation is 421.1 mm, mainly occurring in winter [8].

The study area is located in the Neogene-sedimentary basin of Granada. The dominant substrate comprises lime and gypsum combined with marl, which dates from the late Miocene [60]. The prevailing soils in these outcrops are Leptic gypsisols [61].

The vegetation is predominantly formed of a mosaic of scattered patches of natural plants growing in gypsum outcrops, surrounded by cereal crops, olive groves or almond yards. More precisely, the target habitat for this study, the gypsum habitat, is included in the European Habitat Directive (92/43/EEC) as 1520, “Iberian gypsum vegetation, Gypsophiletalia” [6], and is characterised by three exclusive species of gypsum outcrops (gypsophiles), one of which is a local endemism, *Ononis tridentata* subsp*. crassifolia,* and the other two, *Helianthemum squamatum* and *Lepidium subulatum,* are widespread in the gypsum outcrops of the Iberian Peninsula [8]. In addition, there are also other frequent non-exclusive species of gypsum outcrops (gypsovags) such as *Stipa tenacissima* L., *Helianthemum syriacum* (Jacq.) Dum.Cours., *Helianthemum violaceum* (Cav.) Pau., *Thymus zygis* L. subsp. *gracilis* (Boiss.) R.Morales, *Teucrium capitatum* L. subsp. *gracillimum* (Rouy) Valdés Berm. And Sánchez Crespo*, Rosmarinus officinalis, Hippocrepis bourgaei* (Nyman) Hervier, and *Fumana thymifolia* (L.) Spach Ex Webb [62].

Our experimental site was set on an old cereal field composed of marls, and where, in 2009, post-quarrying conditions were recreated for previous experiments [8] in 20 plots of 5 m × 5 m, using gypsum spoil (rocky waste after gypsum mining; see its physicochemical characterisation in Supplementary Table S4) as the bedding material. Afterwards, seeds of seven gypsum species were sown there in November 2009, including both gypsophiles (*O. tridentata* subsp. *crassifolia, H. squamatum,* and *L. subulatum*) and gypsovags (*S. tenacissima*, *H. syriacum*, *T. zygis* subsp. *gracilis*, and *R. officinalis*), the so-called “key species” in this study. As for experimental plots for this investigation, we selected those plots which currently present comparable soil conditions, such as those with the “sowing” (S) and “sowing plus organic matter addition” (SO) treatments (5 plots each) [8].

### 4.2. Experimental Design

To test whether rosemary interferes with the emergence of other plant species in gypsum habitats, the experiment was designed as follows: within the referred 10 experimental plots, we randomly established 120 circular subplots of 50 cm in diameter; ninety subplots contained one individual of *R. officinalis* in their centres (target rosemary plant), and the remaining 30 were the control subplots, also named “Bare Soil” (BS), which had never had any rosemary plants beforehand. The 90 circular subplots with rosemary plants were subsequently divided into 3 different groups of 30 subplots according to the following treatments: 1. “*Rosmarinus officinalis*” (RO): circular subplots containing one rosemary plant individual at its centre, and under which there was an organic matter layer of its own fallen leaves; 2. “Litterfall” (LF): the central *R. officinalis* plant and any other perennial plants were removed, but fallen leaves were kept; 3. “Rosemary Removal” (RR): both *R. officinalis* plants and fallen leaves were removed, although the soil seed bank was preserved. In summary, 30 replicates of each treatment were randomly applied in circular sampling subplots (30 replicates x 4 treatments = 120 circular subplots).

Afterwards, the seeds of three gypsophile species (*O. tridentata* subsp. *crassifolia*, *H. squamatum* and *L. subulatum*) were manually sown in these 120 circular subplots in December 2014. For this purpose, 120 seeding packages were prepared with seeds that had been manually harvested in the surrounding habitat and then cleaned and stored in paper bags under room conditions until sowing. Each one contained 420 seeds of gypsophile species (200 seeds of *L. subulatum*, 200 seeds of *H. squamatum* and 20 seeds of *O. tridentata* subsp*. crassifolia)*. In total, 50,400 seeds were sown. Few *Ononis* seeds were used due to the low availability in the populations [9].

Moreover, acting as sowing controls, we also established 60 extra subplots within the experimental area with (NSR) and without (NS) rosemary influence, 30 subplots each, where sowing was not accomplished.

### 4.3. Sampling

#### 4.3.1. Seedling Emergence

Seven months after sowing, we recorded the seedling emergence (number and identity) of all perennial plant species in the 120 sown subplots and in the 60 non-sown subplots with the same circular quadrat (50 cm in diameter). Considering the fact that there were no donor plants in the vicinity of the target rosemary plants except for *R. officinalis*, all the emerged seedlings of the other key species must have come from the sowing. Afterwards, we calculated: (1) Overall emergence: total number of emerged seedlings of any perennial species within the experimental subplots; (2) Emergence of key species: total number of seedlings in the group consisting of *O. tridentata* subsp. *crassifolia, H. squamatum, H. syriacum, L. subulatum, R. officinalis, T. zygis* subsp. *gracilis*, and *S. tenacisima*; (3) Emergence of sown species: total number of seedlings in the group consisting of *O. tridentata* subsp. *crassifolia, H. squamatum* or *L. subulatum*; (4) Emergence per key species: total number of seedlings per key species (*O. tridentata* subsp. *crassifolia, H. squamatum, H. syriacum, L. subulatum, R. officinalis, T. zygis* subsp. *Gracilis,* and *S. tenacisima*).

#### 4.3.2. Terpene Content

In this study, terpene composition in soil and leaf essential oils was analysed as “overall composition”, meaning all terpenes present in our samples, and as “key terpenes”, meaning the list of those described as terpenes with allelopathy in the literature [20,21,33,34,35], such as α- and β-pinene, camphor, borneol, carvacrol, thymol, eucalyptol, limonene, o-cymenone, citronellol, camphene, α-phellandrene, and p-cymene. Those terpenes among the previous list found in our samples have been classified as “key terpenes”.

For this purpose, we randomly collected 5 samples of fresh green leaves (GL), 5 samples of fallen leaves (FL), 5 samples of soil from the rosemary plant canopy (RS), and 5 samples of bare (rosemary-free) soil (BS), within the experimental plots. Each sample of leaves comprised c. 75 g of leaves, and each sample of soil was c. 2 kg. Soil and fallen leaf samples were collected in December 2014, whereas fresh green leaf samples were collected in May 2015 for phenological reasons.

In order to preserve the leaf terpenes, fresh leaves were kept in plastic bags in a refrigerator at 5 °C, and fallen leaves were stored in plastic bags under room conditions (c. 25 °C), until the extraction of essential oils was conducted. No further preparation was required.

Soil samples were first spread on filter paper and air dried at room temperature (c. 25 °C) for seven days. Each soil sample was then ground and sieved at 2 mm to remove rocks and other impurities. Finally, each soil sample was placed in a plastic bag and stored in a dry room at 25 °C until the extraction of essential oils was completed.

### 4.4. Laboratory Analysis

The chemical analysis of the essential oils (EOs) present in the soil and leaf samples was oriented to test whether the EOs of the rosemary plants in the study area contained any of the terpenes previously described as inhibitors or retardants of seed germination [20,21,33,34,35], which are listed in Section 4.3.2.

The essential oils in the leaf samples were extracted (three days after leaf collection, so they were still fresh) by a 2 h hydrodistillation process [21], through a Clevenger-like apparatus (700 mL capacity) supplied by the Centre for Scientific Instrumentation (CIC) at the University of Granada, Spain. We used a total of 400 mL of distilled water and 25 g of fresh leaves. The condensed essential oils were collected with a Pasteur pipette and transferred into a vial with 2 mL capacity, and the residue was also captured with a Pasteur pipette after the addition of 2 mL of diethyl-ether. Both vials were kept in the fridge at 5 °C. The diluted residue was used for the chromatographic analysis. The same procedure was repeated with the 5 samples of fresh leaves, and the 5 samples of fallen leaves.

To extract essential oils from soil samples, we used a universal organic solvent such as chloroform [63] as the extractant, since the method used for leaves was not effective with the soil samples. It consisted in putting 200 mL of chloroform stabilised with ethanol (Scharlau Multisolvent HPLC grade ACS ISO UV-VIS) in a 500 mL Erlenmeyer flask, adding 200 g of soil sample, and finally shaking this mixture for 1 h at room temperature with the help of a magnetic stirrer. A watch glass was placed on the top to prevent evaporation in the meantime. After that time, the mixture was filtered through a filter paper and collected in a 250 mL glass beaker (a watch glass was placed on the top to keep away impurities), which was left at room temperature for several days until all the liquid phase was fully evaporated. Then, the residue was resuspended in 1 mL of chloroform and transferred to a vial, which was kept in the fridge at 5 °C until analyses were accomplished. The same procedure was repeated for each soil sample (10 times in total, 5 times per soil sample type).

The content of the vials (with soil or leaf extracts) was analysed (1 mL) through a high-performance liquid chromatography (HPLC). Analyses were accomplished with an Agilent 7890 High Resolution Gas Chromatograph (GC), which was equipped with a Waters Quattro micro™ GC triple quadruple mass spectrometer (MSD). Gas chromatography specifications were: Phenomenex ZB-5MS capillary columns with non-polar phases (30 m × 0.25 mm ID × 0.25 µm film); split ratio (100:1); carrier gas, helium (1 mL/min); 220 °C for both the injector and the transfer line; and working temperatures as follows: from 50 °C (2.5 min) to 200 °C (8 min), with a change rate of 4 °C/min. MSD specifications were: 240 °C as the temperature source; 45 Da–450 Da Fullscan; electron bombardment ionisation (EI+) at 70 eV.

### 4.5. Statistical Analysis

All statistical analyses were performed using R software [64,65]. We assessed the effects of the treatments (BS, RO, LF, and RR) over the emergence (general emergence, emergence of key species, emergence of sown species, and emergence per key species) by applying generalised linear mixed models (GLMMs), through the “lme4” package [66], assuming a Poisson error distribution and log link function. We included treatments as fixed factor, and plots as random factor. Model suitability was assessed by the graphical exploration of residuals [67]. Pairwise comparisons between the treatments in terms of seedling emergence were performed with Tukey’s post hoc tests using a “multcomp” package [68]. Graphs and confidence intervals were obtained with R “ggplot2” v3.3.5 [69]. Mean values and standard deviation were calculated using a ddply function (R “plyr” package, v1.8.6) [70].

To assess the terpene content in treatments, a permutational multivariate analysis of variance using distance matrices (ADONIS) was applied by means of “vegan” package v 2.5–7 [71]. Moreover, to assess the content of “key terpenes” in treatments, we performed a permutational analysis of variance using “lmPerm” package [72].

## 5. Conclusions

Contrary to what was expected, this study confirmed that *Rosmarinus officinalis* adult plants act as a nurse species in the early stages of plant development. In fact, a positive effect of rosemary plants was detected on the emergence of gypsum species, especially on the emergence of key species, suggesting that there are ongoing facilitation processes.

However, we found allelopathic effects in relation to rosemary leaves. In this sense, we identified a wide variety of terpenes composing the essential oils of rosemary leaves (both green and fallen) and soils under rosemary plants, most of which are common to all. Consequently, rosemary leaves seem to release terpenes to the neighbouring soils, which has proven to be a determining factor of seedling emergence, probably due to the fact that the most abundant terpenes have shown strong inhibitory effects in previous studies. Hence, in the treatment in which rosemary plants were removed but the organic layer was conserved, a much lower emergence was registered in comparison to the other three treatments, underlining a significant allelopathic effect.

In relation to the role of *R. officinalis* in the recovery of gypsum habitats, in those areas in which *R. officinalis* is highly dominant, we do not suggest removing it in the early stages of plant development in order to encourage seedling emergence. Conversely, this could be the right technique to apply later to stimulate other stages of a plant life cycle, such as survival or growth, since a high content of terpenes, combined with competition for light, water and/or nutrients, could negatively affect plant growth and therefore, condition plant diversity recovery in degraded gypsum habitats.

In brief, restoration plans for degraded gypsum habitats should promote facilitation interactions between nurse plants and the target species, whereas allelopathy should be avoided.

## Figures and Tables

**Table 1:** Mean (+SE) emergence (nº of seedlings) per species and treatment. Different letters represent statistically significant differences (*p* < 0.05) for the post hoc Tukey tests performed after the GLMMs, where “a” represents the lowest value and “d” the highest. Treatments: BS: Bare Soil; LF: Litterfall; RR: Rosemary Removal; RO: *Rosmarinus officinalis*.

Key Species	Emergence (Mean + SE) Per Treatment			
	RO	LF	RR	BS
*O. tridentata* subsp. *crassifolia*	0.13 ± 0.10 a	0.13 ± 0.08 a	0.07 ± 0.04 a	0.13 ± 0.06 a
*H. squamatum*	6.03 ± 1.25 d	0.67 ± 0.19 a	3.50 ± 0.71 c	2.23 ± 0.39 b
*L. subulatum*	0.13 ± 0.08 ab	0.03 ± 0.03 a	0.07 ± 0.05 a	0.50 ± 0.20 b
*R. officinalis*	1.4 ± 0.89 b	0.13 ± 0.08 a	0.10 ± 0.07 a	0.10 ± 0.06 a
*H. syriacum*	0.17 ± 0.11 ab	0.00 ± 0.00 a	0.03 ± 0.03 a	0.23 ± 0.08 b
*T. zygis* subsp. *gracilis*	2.03 ± 0.79 b	0.97 ± 0.29 a	0.53 ± 0.24 a	1.03 ± 0.20 a
*S. tenacissima*	0.00 ± 0.00 a	0.00 ± 0.00 a	0.10 ± 0.07 a	0.00 ± 0.00 a

**Table 2:** Presence of terpene compounds identified by GC–MS in the essential oil of *R. officinalis* leaf and soil samples. “X” means “presence of terpene”. GL: green leaves; FL: fallen leaves; BS: bare soil; RS: soil under rosemary plant.

N	Terpene Compound	Retention Time (Seconds)	Key Terpene	Sample Types			
				Leaves		Soils	
				GL	FL	BS	RS
1	tricyclene	7.34	No		X		X
2	thujene	7.46	No	X			
3	α-pinene	7.59–7.78	Yes	X	X		X
4	camphene	8.11–8.27	Yes	X	X		X
5	verbenene	8.22	No				X
6	β-pinene	9.03–9.19	Yes	X	X		X
7	1-octen-3-ol	9.21–9.27	No		X		
8	3- octanone	9.34–9.45	No		X		
9	β-myrcene	9.51–9.65	No	X	X		X
10	3- heptanol	9.86	No		X		
11	α-phellandrene	10.07–10.18	Yes		X		X
12	artemisatriene	10.45–10.56	No				X
13	p-cymene	10.74–10.84	Yes	X	X		X
14	limonene	10.94–11.05	Yes	X	X		X
15	eucalyptol (1’8 cineole)	11.04–11.16	Yes	X	X		X
16	ɤ_terpinene	11.97–12.06	No	X			X
17	terpinolene	12.97–13.04	No	X			
18	linalool	13.51–13.61	No	X	X		X
19	3.5 heptadien-2-ol-2.6	14.26–14.33	No	X	X		X
20	camphenol	14.45–14.53	No				X
21	pinocarveol	14.99–15.13	No				X
22	camphor	15.17–15.36	Yes	X	X		X
23	iso-pinocamphone	15.66–15.73	No				X
24	borneol	16.10–16.21	Yes	X	X		X
25	terpinen-4-ol	16.41–16.49	No	X	X		X
26	α-terpineol	16.97–17.02	No	X	X		X
27	α-santolin-alcohol	17.30	No	X			
28	verbenone	17.42	No	X	X		X
29	bornyl acetate	20.04	No	X			
30	carvacrol	20.3	Yes				X
31	copaene	23.11	No				X
32	caryophyllene	24.52–24.61	No	X	X		X
33	aromadendrene	25.63	No	X	X		
34	cis-α-bisabolene	25.715	No	X	X		X
35	methyl 8′11′14′17-eicosatetraenoate	27.544	No	X			
36	caryophyllene oxide	29.42	No	X	X		X
37	farnesol	29.51–30.00	No				X
38	tridecan	31.05	No				X
39	ledene oxyde	31.58	No				X

**Table 3:** Significant differences (*p* < 0.05) between sample types for the post hoc test performed after multivariate analysis. Significant codes: ‘*’ 0.05. GL: green leaves; FL: fallen leaves; RS: soil under rosemary plant.

Pairs	Df	SumsOfSqs	F.Model	R2	*p*. Value	*p*. Adjusted	Sig
FL vs. GL	1.00	0.27	4.92	0.38	0.01	0.02	*
FL vs. RS	1.00	0.13	1.17	0.13	0.32	0.97	
GL vs. RS	1.00	0.33	3.55	0.31	0.01	0.02	*

**Table 4:** Significant differences between the sample types per key terpene. Different letters represent statistically significant differences (*p* < 0.05) for the post hoc test performed after lmPerm, where “a” represents the lowest value and “c” the highest. GL: green leaves; FL: fallen leaves; RS: soil under rosemary plant; BS: bare soil.

Terpenes	Sample Types			
	Leaves		Soils	
	GL	FL	BS	RS
α-pinene	b	b	a	b
camphene	b	b	a	b
β-pinene	c	ab	a	bc
α-phellandrene	a	a	a	a
p-cymene	ab	b	a	ab
limonene	b	ab	a	ab
eucalyptol	b	b	a	b
camphor	b	b	a	b
borneol	b	b	a	b
carvacrol	a	a	a	a

**Table 5:** Summary of the main characteristics of treatments and their effect over emergence. “**+**” = positive effect; “**−**“ = negative effect. Treatments: RO: *Rosmarinus officinalis*; RR: Rosemary Removal; LF: Litterfall; BS: Bare Soil.

Treatments	Rosemary Plant Presence	Rosemary Leaves Presence	Allelopathic Compounds	Facilitation Effect	Effect over Emergence
RO	Yes	Yes	Yes	Yes	+++
LF	Removal	Yes	Yes	No	--
RR	Removal	Removal	Residual	No	+
BS	Never	Never	No	No	+

## Data Availability

The data presented in this study are openly available in FigShare at https://doi.org/10.6084/m9.figshare.19127858.

## Supplementary Materials

The following supporting information can be downloaded at: https://doi.org/10.6084/m9.figshare.19127858, Table S1: Effect of surface treatment on overall seedling emergence; Table S2: List of terpenes in the essential oil from soil and leaf samples; Table S3: Relative abundance of terpenes; and Table S4: Mean values of the physicochemical characterisation of gypsum spoil.

## Abbreviations

RO = *Rosmarinus officinalis*; RR = Rosemary Removal; LF = Litterfall; BS **=** Bare Soil; RS = Rosemary Soil; FL **=** Fallen Leaves; GL = Green Leaves.
